# Estimated Reduction in Cancer Risk due to PAH Exposures If Source Control Measures during the 2008 Beijing Olympics Were Sustained

**DOI:** 10.1289/ehp.1003100

**Published:** 2011-02-08

**Authors:** Yuling Jia, Dave Stone, Wentao Wang, Jill Schrlau, Shu Tao, Staci L. Massey Simonich

**Affiliations:** 1 Environmental and Molecular Toxicology, Oregon State University, Corvallis, Oregon, USA; 2 College of Urban and Environmental Science, Peking University, Beijing, China; 3 Department of Chemistry, Oregon State University, Corvallis, Oregon USA

**Keywords:** air pollution, Beijing Olympics, cancer risk assessment, China, megacity, PAH, PM_2.5_, polycyclic aromatic hydrocarbon

## Abstract

**Background:**

The 2008 Beijing Olympic Games provided a unique case study to investigate the effect of source control measures on the reduction in air pollution, and associated inhalation cancer risk, in a Chinese megacity.

**Objectives:**

We measured 17 carcinogenic polycyclic aromatic hydrocarbons (PAHs) and estimated the lifetime excess inhalation cancer risk during different periods of the Beijing Olympic Games, to assess the effectiveness of source control measures in reducing PAH-induced inhalation cancer risks.

**Methods:**

PAH concentrations were measured in samples of particulate matter ≤ 2.5 μm in aerodynamic diameter (PM_2.5_) collected during the Beijing Olympic Games, and the associated inhalation cancer risks were estimated using a point-estimate approach based on relative potency factors.

**Results:**

We estimated the number of lifetime excess cancer cases due to exposure to the 17 carcinogenic PAHs [12 priority pollutant PAHs and five high-molecular-weight (302 Da) PAHs (MW 302 PAHs)] to range from 6.5 to 518 per million people for the source control period concentrations and from 12.2 to 964 per million people for the nonsource control period concentrations. This would correspond to a 46% reduction in estimated inhalation cancer risk due to source control measures, if these measures were sustained over time. Benzo[*b*]fluoranthene, dibenz[*a*,*h*]anthracene, benzo[*a*]pyrene, and dibenzo[*a*,*l*]pyrene were the most carcinogenic PAH species evaluated. Total excess inhalation cancer risk would be underestimated by 23% if we did not include the five MW 302 PAHs in the risk calculation.

**Conclusions:**

Source control measures, such as those imposed during the 2008 Beijing Olympics, can significantly reduce the inhalation cancer risk associated with PAH exposure in Chinese megacities similar to Beijing. MW 302 PAHs are a significant contributor to the estimated overall inhalation cancer risk.

Outdoor air pollution is a significant health concern in China (Chan and Yao 2008; Kan et al. 2009). In 2003, the estimated health care costs associated with outdoor air pollution in China ranged from 157 billion renminbi (RMB) to 520 billion RMB, accounting for 1.2–3.8% of China’s gross domestic product (World Bank 2007). An estimated 300,000 people die each year in China from heart disease and lung cancer associated with exposure to ambient air pollution, including carcinogenic polycyclic aromatic hydrocarbons (PAHs) (Ishikawa et al. 2006; Kahn and Yardley 2007; Mumford et al. 1987). Specifically, an annual average of 6.5 per million people in China are estimated to have lung cancer due to PAH inhalation exposure, and populations in major Chinese cities have a higher risk of lung cancer than average for China (Zhang Y et al. 2009). PAH emissions from biomass and coal combustion for heating and cooking are a dominant source of indoor air pollution in China and likely contribute a significant portion to the overall cancer risk due to PAH exposure in the Chinese population (Zhang and Smith 2007).

Chinese megacities, including Beijing, suffer from severe air pollution due to increasing coal combustion and motor vehicle emissions (Chan and Yao 2008). PAH concentrations higher than China’s national ambient PAH standards (China State Environmental Protection Agency 1996) have been measured in Beijing’s ambient particulate matter (PM) (Liu L-B et al. 2007; Liu Y et al. 2007; Okuda et al. 2006; Zhang S et al. 2009), and an estimated 1.7% of cancers diagnosed in Beijing residents during 1997 (~ 272–309 cases) were attributable to PAH pollution in the air (Yu et al. 2008).

The Beijing government made an unprecedented effort to improve air quality for the 2008 Beijing Olympic Games (8–24 August 2008). Source control measures carried out from 20 July to 20 September 2008 included moving or closing pollution-emitting factories in the city and neighboring municipalities, restricting vehicles on alternate days under an even–odd license plate system, and reducing heavy-duty truck traffic (Wang et al. 2010; Wang WT et al. 2009; Wang X et al. 2009; Zhang XY et al. 2009). More stringent control measures, including decreased coal combustion, were implemented during the Olympic Games (Zhang XY et al. 2009). It has been estimated that the traffic volume of typical roads in Beijing was reduced by approximately 32% during the source control period (Shou-bin et al. 2009; Wang and Xie 2009; Wang et al. 2010). Emissions from the power plants, coal-fired boilers, and several heavy-polluting factories in Beijing were reduced by 30–50% during the Olympic Games (Wang et al. 2010). Consequently, significant reductions in PM concentrations were measured during the Olympic Games (Wang WT et al. 2009; Wang X et al. 2009). In addition, Li et al. (2010) reported a significant reduction in adult asthma hospital visits during the Olympic Games, and Wu et al. (2010) reported evidence of altered cardiac function (heart rate variability)–associated PM_2.5_ concentrations in a study of Beijing taxi drivers before, during, and after the Olympic Games.

The health benefits associated with air pollution emission reductions have recently been assessed in the highly populated Yangtze River Delta region of China (Zhou et al. 2010). Likewise, the 2008 Beijing Olympic Games provided a unique case study to understand the change in inhalation cancer risk associated with PAH exposure in one of China’s three megacities with the implementation of source control measures. In this study, we measured the ambient concentrations of 17 carcinogenic PAHs on PM_2.5_ (PM with aerodynamic diameter ≤ 2.5 μm) collected before, during, and after the 2008 Beijing Olympic Games and estimated the associated excess inhalation cancer risk during the different periods. Of the 17 carcinogenic PAHs measured, 12 are U.S. Environmental Protection Agency (EPA) priority pollutants, and five are high-molecular-weight (302 Da) PAHs (MW 302 PAHs). Four of the five MW 302 PAHs were dibenzopyrene isomers that have been shown to be highly carcinogenic (Boström et al. 2002; Layshock et al. 2010; Straif et al. 2005). Despite their high carcinogenicity, MW 302 PAHs are rarely studied because of the lack of commercial standards and their low environmental concentrations compared with the priority pollutant PAHs (Fetzer 2000). The goals of this study were to estimate the excess inhalation cancer risk during source control (28 July to 20 September), nonsource control (21 September to 7 October), Olympic (8–24 August), and non-Olympic periods (28 July to 7 August and 25 August to 7 October) to assess the effectiveness of source control measures in reducing PAH-induced inhalation cancer risks in a Chinese megacity and to compare the estimated inhalation cancer risk posed by MW 302 PAHs with that posed by conventional priority pollutant PAHs. To our knowledge, this is the first report to examine changes in estimated inhalation cancer risk reduction as a result of source control strategies in the Chinese population.

## Materials and Methods

### Sampling

The sampling location and sample collection have been previously described (Wang WT et al. 2009). Briefly, sampling was conducted on the roof of the Geology Building (~ 25 m above ground) on the Peking University campus in Beijing from 28 July to 3 September 2008 and from 13 September to 7 October 2008. Source control measures were carried out by the Beijing government from 20 July to 20 September 2008 (Wang et al. 2010; Wang WT et al. 2009; Wang X et al. 2009; Zhang XY et al. 2009). Samples were not collected from 4 to 12 September 2008 because of sampler motor failure, and these days were excluded from the calculation of cancer risks.

PM_2.5_ samples were collected on prebaked (350°C) quartz fiber filters (Tisch Environmental Inc., Cleves, OH, USA) using a high-volume air sampler coupled with a three-stage Cascade Impactor (TE-230; Tisch Environmental Inc.). Sampling operation was in accordance with procedures established by U.S. EPA (1975) and ASTM Specification D2009 (Wang WT et al. 2009). A total of 63 24-hr PM_2.5_ samples were collected along with six field blanks. More details of the sampling can be found in Wang WT et al. (2009).

### Sample extraction

The sample extraction method used in this study has previously been described in detail (Primbs et al. 2007, 2008a, 2008b). Briefly, the PM_2.5_ sample filters were spiked with perdeuterated surrogate standards (d_10_-fluorene, d_10_-phenanthrene, d_10_-pyrene, d_12_-triphenylene, d_12_-benzo[*a*]pyrene, and d_12_-benzo[*g*,*h*,*i*]perylene) and extracted twice with dichloromethane (Optima; Fisher Scientific, Hampton, NH, USA) using pressurized liquid extraction (ASE 300; Dionex Corp., Sunnyvale, CA, USA) (Primbs et al. 2008a). The extracts were combined and eluted with 100 mL dichloromethane through a 20-g silica column [Varian Inc. (Agilent Technologies), Santa Clara, CA, USA] that was preconditioned with ethyl acetate, dichloromethane, and hexane. Internal PAH standards (d_10_-acenaphthene, d_10_-fluoranthene, and d_12_-benzo[*k*]fluoranthene) were then added to the purified extract before analysis using gas chromatography/mass spectrometry (GC/MS).

### PAH measurement

The sample extract was analyzed by GC (Agilent 5890 GC; Agilent Technologies, Santa Clara, CA, USA) coupled to a quadrupole MS using electron impact ionization (70 eV; Agilent 5973 MSD). Measurement of the U.S. EPA priority pollutant PAHs was achieved using a DB-5MS column and has been previously described in detail (Primbs et al. 2007, 2008a, 2008b). Measurement of MW 302 PAHs was achieved using a 50% phenyl DB-17MS column [60 m × 0.25 mm × 0.25 μm; J&W Scientific (Agilent Technologies), Folsom, CA, USA], which is able to separate dibenzo[*a*,*l*]pyrene from the other MW 302 PAH isomers (Sauvain and Vu Duc 2004; Schubert et al. 2003). The GC temperature program was adapted from Schubert et al. (2003): temperature hold at 100°C for 1 min, ramp 40°C/min until 200°C, 2°C/min until 310°C, temperature hold at 310°C for 58 min, and ramp 45°C/min until 320°C, with final isothermal hold at 320°C for 0.5 min. Ion source, quadrupole, and transfer line of the MS were kept at 230°C, 150°C, and 300°C, respectively. Compound identification and verification was performed by GC/MS in the scan mode (100–350 *m*/*z*), whereas quantitative analysis of all samples was performed in selected ion monitoring mode.

### Risk assessment

A point-estimate approach, developed by the Office of Environmental Health Hazard Assessment (OEHHA) of the California Environmental Protection Agency (CalEPA) (Collins et al. 1998; OEHHA 2003), was used to estimate the inhalation cancer risk of PAHs during the different periods. The benzo[*a*]pyrene (BaP) equivalent (BaP_eq_) concentration of the individual PAHs was calculated by multiplying their concentration by their relative potency factor (RPF). The inhalation cancer risk for the PAH mixture was estimated by multiplying the sum of the individual BaP_eq_ concentrations by the unit risk (UR) of exposure to BaP. The equation for this calculation is as follows:


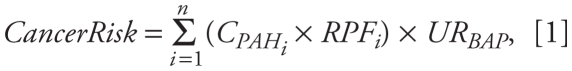


where *C_PAHi_* is the concentration of the *i*th individual PAH, *n* is the number of individual PAHs measured in air, *RPF_i_* is the RPF of the *i*th individual PAH, and *UR_BaP_* is the inhalation UR of exposure to BaP (specifically, “the calculated, theoretical upper limit possibility of contracting cancer when exposed to BaP at a concentrations of one microgram per cubic meter of air for a 70-year lifetime”) (OEHHA 1993, 2005). CalEPA has calculated a UR_BaP_ estimate of 1.1 × 10^−6^ per ng/m^3^ based on the data for respiratory tract tumors from inhalation exposure in hamsters (OEHHA 1993, 2005), whereas the World Health Organization (WHO) has estimated UR_BaP_ at 8.7 × 10^−5^ per ng/m^3^ based on an epidemiology study on coke-oven workers in Pennsylvania (WHO 2000). In this study, both UR_BaP_ values were used to calculate excess PAH-induced inhalation cancer risk.

Seventeen carcinogenic PAHs were included in the estimate of inhalation cancer risk, including 12 priority pollutant PAHs [anthracene (ANT), benz[*a*]anthracene (BaA), benzo[*b*]fluoranthene (BbF), benzo[*g,h,i*]perylene (BghiP), benzo[*k*]fluoranthene (BkF), chrysene (CHR), dibenz[*a*,*h*]anthracene (DahA), fluoranthene (FLA), indeno[1,2,3-*cd*]pyrene (IcdP), phenanthrene (PHE), pyrene (PYR), and benzo[*a*]pyrene (BaP)] and 5 MW 302 PAHs [dibenzo[*a*,*e*]pyrene (DBaeP), dibenzo[*a*,*h*]pyrene (DBahP), dibenzo[*a*,*i*]pyrene (DBaiP), dibenzo[*a*,*l*]pyrene (DBalP), and naphtho[2,3-*e*]pyrene (N23eP)] (Table 1). Because CHR coeluted with triphenylene (TRI), a ratio of CHR:TRI = 2.4 [their ratio in the National Institute of Standards and Technology (NIST) SRM 1649b urban dust standard; NIST 2009] was used to estimate the CHR concentration. Other measured PAHs were not included in the estimate of inhalation cancer risk because of the lack of RPF values for these PAHs. RPF values based on tumor bioassay data from the U.S. EPA’s Integrated Risk Information System Program were used (U.S. EPA 2010). RPF values for additional MW 302 PAH isomers were also reviewed by U.S. EPA; however, these values were not selected by the U.S. EPA because of the inadequacy of the data for these isomers (U.S. EPA 2010). Table 1 lists the individual PAHs and their respective RPF values. An alpha level of 0.05 is considered statistically significant throughout this article.

## Results and Discussion

### BaP_eq_ concentrations and effect of source control

The BaP_eq_ concentrations of the individual PAHs, the sum of the 12 priority pollutant PAHs (∑PPAH_12-BaPeq_), the sum of the five MW 302 PAHs (∑302PAH_5-BaPeq_), and the sum of the 17 PAHs (∑PAH_17-BaPeq_) during the source control and nonsource control periods are given in Supplemental Material, Table 1 (doi:10.1289/ehp.1003100); their concentrations during the Olympic and non-Olympic periods are given in Supplemental Material, Table 2. The average total concentration of the 12 priority pollutant PAHs decreased from a measured value of 11.4 ng/m^3^ to 6.1 ng/m^3^ after conversion to their BaP_eq_ concentration. In contrast, the average total concentration of the five MW 302 PAHs increased from a measured value of 0.47 ng/m^3^ to 1.24 ng/m^3^ after conversion to their BaP_eq_ concentration (see Supplemental Material, Figure 1).

**Figure 1:**
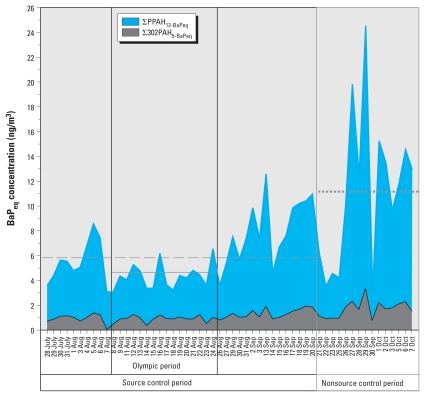
Temporal variation of ∑PPAH_12-BaPeq_ and ∑302PAH_5-BaPeq_. Horizontal lines show ∑PAH_17-BaPeq_ during the source control period (dashed line), the nonsource control period (dotted line), and the Olympic period (solid line). The figure excludes 4–12 September 2008 because of sampler motor failure.

Figure 1 shows the temporal variation of total BaP_eq_ concentration of the 17 carcinogenic PAHs throughout the entire sampling period. The daily estimated ∑PAH_17-BaPeq_ ranged from 3.1 to 24.5 ng/m^3^ during the sampling period, with average concentrations of 5.95 ng/m^3^, 11.1 ng/m^3^, 4.33 ng/m^3^, and 11.7 ng/m^3^ during the source control, nonsource control, Olympic, and non-Olympic periods, respectively. China’s State Environmental Protection Agency’s (1996) daily BaP_eq_ standard (10 ng/m^3^) was exceeded on 13 of the 56 sampling days (12 of these days were during the nonsource control period). In addition, all sampling days exceeded the European Union’s annual average BaP_eq_ standard (1 ng/m^3^) (European Commission 2001).

The BaP_eq_ concentrations in Beijing have been previously estimated using a toxic equivalency factor (TEF) approach. Liu Y et al. (2007) reported an average BaP_eq_ concentration of 13.0 ng/m^3^ and 27.3 ng/m^3^ for 16 priority pollutant PAHs in the winter of 2005 and < 1 ng/m^3^ in the summer of 2004 at two sampling sites on the Peking University campus. Zhang S et al. (2009) estimated a particle-phase BaP_eq_ concentration of approximately 2.5 ng/m^3^ for 16 priority pollutant PAHs in a southeast suburb of Beijing in the summer of 2005. Layshock et al. (2010) reported BaP_eq_ concentrations for seven priority pollutant PAHs and four dibenzopyrene isomers of 2.4 ng/m^3^ in the summer of 2007 and 231 ng/m^3^ in the winter of 2008. Our estimated BaP_eq_ concentrations, based on the most recently recommended RPF approach (U.S. EPA 2010), are slightly higher than previous studies that were based on the TEF approach. This is because of the difference in the RPF and TEF values, inclusion of different PAHs in the assessments, and variation in PAH concentrations.

We estimated statistically significant BaP_eq_ concentration reductions (*p* ≤ 0.01) for all individual PAHs during the source control period relative to the nonsource control period, with estimated concentration reductions ranging from 27% to 78% [see Supplemental Material, Table 1 (doi:10.1289/ehp.1003100)]. ∑PPAH_12-BaPeq_ was reduced from 9.4 ± 5.5 ng/m^3^ to 4.9 ± 2.1 ng/m^3^ (a reduction of 48%, *p*-value < 0.001), whereas ∑302PAH_5-BaPeq_ was reduced from 1.7 ± 0.7 ng/m^3^ to 1.1 ± 0.4 ng/m^3^ (a reduction of 38%, *p* < 0.001). ∑PAH_17-BaPeq_ was reduced by 46% (*p* < 0.001) from the nonsource control period (11.1 ± 6.2 ng/m^3^) versus the source control period (6.0 ± 2.5 ng/m^3^). We observed similar reductions during the Olympic period compared with the non-Olympic period, with BaP_eq_ concentrations estimated for individual PAH reduced by 32% to 72% and estimated summed concentrations reduced by 52% (*p* < 0.001), 34% (*p* = 0.002), and 49% (*p* < 0.001) for ∑PPAH_12-BaPeq_, ∑302PAH_5-BaPeq_, and ∑PAH_17-BaPeq_, respectively (see Supplemental Material, Table 2). These differences likely resulted from the implementation of more stringent control measures during the Olympic Games, especially for coal combustion (Zhang XY et al. 2009), but favorable meteorological conditions may also have contributed to reduced PM concentrations during the Olympic Games compared with the rest of the source control period (Wang WT et al. 2009; Zhang XY et al. 2009).

**Figure 2:**
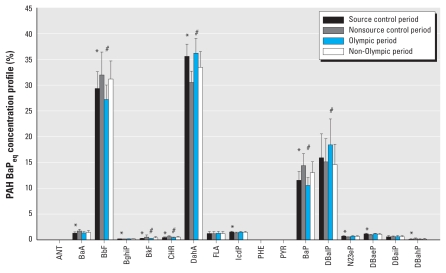
Mean ± SD BaP_eq_ concentration profile of the individual PAHs during the source control, nonsource control, Olympic, and non-Olympic periods. *p < 0.05 between source control and nonsource control periods; ^#^*p* < 0.05 between Olympic and non-Olympic periods.

Despite the significant reduction in estimated BaP_eq_ concentrations, proportional contributions of the 17 PAHs to the overall BaP_eq_ concentration profile were similar between the source control and nonsource control periods and between the Olympic and non-Olympic periods (Figure 2). BaP_eq_ concentrations only for BbF, BkF, CHR, DahA, and BaP were statistically significantly different between the source control and nonsource control periods, and the Olympic and non-Olympic periods. This suggests that the source control measures did not greatly change the composition of carcinogenic PAHs in PM_2.5_.

The ∑PPAH_12-BaPeq_:∑302PAH_5-BaPeq_ ratio was not significantly different between the source control (5.7 ± 7.7) and nonsource control (5.1 ± 1.6) periods (*p* = 0.28) but was significantly different between the Olympic (4.1 ± 1.5) and non-Olympic (6.1 ± 7.7) periods (*p* < 0.01), likely because of the implementation of more stringent source control measures during the Olympic Games (Wang et al. 2010; Zhang XY et al. 2009).

BbF, DahA, BaP, and DBalP accounted for 30%, 34%, 12%, and 16% of ∑PAH_17-BaPeq_, respectively (91–93% of the total combined) (Figure 2). Although BbF and BaP had relatively high concentrations (2.9 ng/m^3^ and 1.0 ng/m^3^ on average, respectively), DahA and DBalP had high RPF values (10 and 30, respectively) compared with the other measured PAHs.

BbF and BaP contributed a slightly higher percentage to ∑PAH_17-BaPeq_ during the nonsource control and non-Olympic periods compared with the source control and Olympic periods (32% vs. 29% and 14% vs. 12%, respectively; *p* < 0.05), but the opposite was true for DahA (31% vs. 36%) (Figure 2). These small differences suggest that the proportion of individual PAHs emitted from sources remained relatively constant, despite changes in overall PAH emissions. Efforts to reduce BbF, DahA, BaP, and DBalP emissions would substantially reduce the total BaP_eq_ concentration in Beijing. Because these PAHs are primarily associated with PM_2.5_, reducing PM_2.5_ emissions would reduce their concentrations, as well as concentrations of other PAHs.

### Estimate of inhalation cancer risk

We used two different UR_BaP_ values in this study to estimate the inhalation cancer risk: One UR_BaP_ value (1.1 × 10^−6^ per ng/m^3^) was derived from a rodent study (OEHHA 1993) and another (8.7 × 10^−5^ per ng/m^3^) was derived from an epidemiology study (WHO 2000). Because of the statistically significant difference in total BaP_eq_ concentrations between the source control and nonsource control periods and between the Olympic and non-Olympic periods, we calculated the inhalation cancer risk estimates and compared them for the different periods.

**Figure 3:**
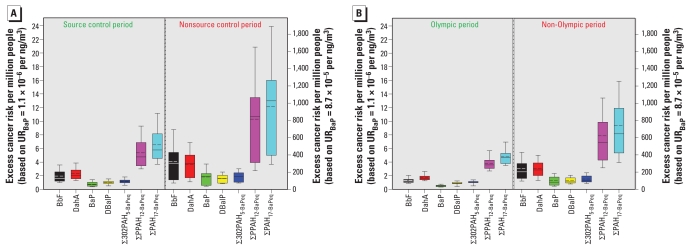
Estimated number of excess inhalation cancer cases for selected PAHs during the source control and nonsource control periods (*A*) and during the Olympic and non-Olympic periods (*B*): left *y*-axes, based on a UR_BaP_ of 1.1 × 10^−6^ per ng/m^3^ from a rodent study (OEHHA 1993); right *y*-axes, based on a UR_BaP_ of 8.7 × 10^−5^ per ng/m^3^ from an epidemiology study (WHO 2000). The boxes represent the 25th and 75th percentiles, the whiskers represent the 10th and 90th percentiles, and the short dash and solid lines within the boxes represent the mean and median, respectively.

Figure 3A shows the estimated lifetime excess inhalation cancer risk per million people due to PAH exposures during source control compared with nonsource control periods. Using a UR_BaP_ value of 1.1 × 10^−6^ per ng/m^3^, the average estimated excess inhalation cancer risk associated with ∑PAH_17-BaPeq_ exposures during the source control period is 6.5 cancer cases per million people in an average lifetime of 70 years, compared with 12.2 per million people for exposures during the nonsource control period. The 12 priority pollutant PAHs were associated with an estimated inhalation cancer risk increase of 5.4 per million people during the source control period and 10.3 per million people during the nonsource control period. The five MW 302 PAHs contributed to an estimated inhalation cancer risk increase of 1.2 and 1.9 per million people for the source control and nonsource control periods, respectively. Of the individual PAHs, BbF and DahA contributed the most to the estimated excess inhalation cancer risk, followed by DBalP and BaP (Figure 3A).

Alternatively, if a UR_BaP_ value of 8.7 × 10^−5^ per ng/m^3^ is used, the average estimated excess inhalation cancer risks due to ∑PAH_17-BaPeq_, ∑PPAH_12-BaPeq_, and ∑302PAH_5-BaPeq_ exposures during the source control period were 518, 425, and 93 per million people, respectively, compared with excess risks of 964, 815, and 149 per million people during the nonsource control period. Similarly, the estimated lifetime excess inhalation cancer risks for the 17 PAHs were significantly lower during the Olympic period compared with the non-Olympic period (Figure 3B). Depending on which UR we used, we estimated the average excess inhalation cancer risk associated with ∑PAH_17-BaPeq_ at 4.8 (UR_BaP_ = 1.1 × 10^−6^ per ng/m^3^) or 377 (UR_BaP_ = 8.7 × 10^−5^ per ng/m^3^) per million people for the Olympic period concentrations, compared with 9.4 (UR_BaP_ = 1.1 × 10^−6^ per ng/m^3^) or 741 (UR_BaP_ = 8.7 × 10^−5^ per ng/m^3^) per million people for the non-Olympic period concentrations.

Our estimates of inhalation cancer risk associated with ∑PPAH_12-BaPeq_ (518 and 964 per million people for the source control and nonsource control periods, or 637 per million when averaged over the entire study period) are similar to the population-weighted excess annual lung cancer risk due to 16 priority PAHs in the general Chinese population (Zhang Y et al. 2009), estimated using a Euler atmospheric transport model and Monte Carlo simulation (annual incidence rate of 6.5 per million people corresponding to 455 cases per million people > 70 years of age). In addition, Liu Y et al. (2007) used a similar approach and estimated a PAH-induced inhalation cancer risk of 583 per million people for Beijing traffic police and 416 per million people for 10% of the high-risk population (accumulative frequencies) in Beijing.

We estimate that the average total excess inhalation cancer risk due to the 17 carcinogenic PAHs would be lower by 46% (source control vs. nonsource control periods) to 49% (Olympic vs. non-Olympic periods) if source control measures were sustained over time. In addition, estimated reductions in the excess inhalation cancer risk were more pronounced in association with reductions in ∑PPAH_12-BaPeq_ versus ∑302PAH_5-BaPeq_ concentrations during the source control (48% vs. 38%) and Olympic periods (52% vs. 34%). This suggests that the source control strategies implemented by the Beijing government had a larger impact on reducing the cancer risks associated with the priority pollutant PAHs than on those associated with the high-molecular-weight PAHs.

No matter which UR_BaP_ value is used, the estimated excess inhalation cancer risk due to ∑302PAH_5-BaPeq_ is 1.6 ± 0.6 times higher than the excess risk due to BaP alone, and the total excess inhalation cancer risk for all PAH exposures combined is 23% higher when the five MW 302 PAHs are included. This suggests that the MW 302 PAHs are a significant component of the carcinogenic PAH mixture in Beijing air, as well as the air in other Chinese megacities similar to Beijing, and should be included in future inhalation cancer risk assessment in addition to the conventionally measured priority pollutant PAHs.

Although outside of the scope of this population-level risk assessment, it is important to note that the response among various subpopulations to the carcinogenic potential of PAHs is not uniform. This differential response can be the result of genetic factors, such as polymorphisms in glutathione *S*-transferases that alter the capacity to metabolize PAH compounds (Hecht 1999) or variants in cytochrome P450 genes (Ishibe et al. 1998). Behavioral factors such as smoking are relevant to individual risk, given the strong association between smoking and PAH–DNA adducts (Shantakumar et al. 2005). Occupation is another important consideration for an individual’s cancer risk due to PAH exposure (Boffetta et al. 1997). The carcinogenic risk to individuals or subpopulations that have genetic, behavioral, or occupational issues that increase their susceptibility to PAHs may be underrepresented by this assessment.

Like previous inhalation cancer risk assessments, this assessment has uncertainties and limitations. First, the point-estimate approach assumes additive cancer risk. This approach may not be entirely accurate, but it is the currently accepted approach for assessing inhalation cancer risk due to PAH exposure (Collins et al. 1998; OEHHA 2003). Second, the RPF values used to calculate the BaP_eq_ concentration for the individual PAHs are estimates based on toxicologic studies that suffer from additional uncertainties. However, we used RPF values that are the most recently accepted estimates based on a comprehensive analysis of tumor bioassay data (U.S. EPA 2010). Third, the inhalation cancer risk models we used assume a lifetime of exposure, whereas the source control measures were implemented for relatively short durations. Thus, to realize any cancer risk reductions, these measures would have to be sustained over time. Finally, we have extrapolated to all of Beijing the PAH concentrations in PM_2.5_ collected from a single, representative site in Beijing.

Numerous meteorologic and PAH source type factors may influence the PAH concentration and profile in different areas of Beijing that would not be reflected in this point-estimate approach. The limitations and assumptions used in this risk assessment may result in an over- or underestimate of risk to the Beijing population.

A key component of the source control measures during the Beijing Olympics was restriction of on-road vehicles, in addition to industrial emission controls. As a result, the traffic volume was reduced by approximately 32% during the source control period (Shou-bin et al. 2009; Wang and Xie 2009; Wang et al. 2010). However, the number of vehicles in Beijing is increasing by 13% per year, with 4 million vehicles in 2009 (Wu et al. 2011). Similarly, vehicle ownership in other Chinese megacities, such as Shanghai, exceeded 2 million in 2004, with an increase of 17% per year (Shanghai Municipal Statistics Bureau 2005). Our study findings, combined with the rapid increase of vehicles (Wu et al. 2011), suggest that, in addition to restrictions on emission from coal combustion sources, controlling vehicle emissions is key to reducing the inhalation cancer risks due to PAH exposure in Chinese megacities similar to Beijing. After the 2008 Olympic Games, the Beijing government restricted vehicles by 1 day/week, for a 2-year demonstration period (Wu et al. 2011). Other strategies for reducing PAH emission from vehicles in Chinese megacities, and reducing the associated inhalation cancer risk, include development and encouragement of public transportation and more stringent vehicle emission standards (Wu et al. 2011).

## Conclusion

Using an RPF approach, we estimated the BaP_eq_ concentration of 17 carcinogenic PAHs before, during, and after the 2008 Beijing Olympics on PM_2.5_. Estimated ∑PAH_17-BaPeq_ concentrations ranged from 3.1 to 12.6 ng/m^3^, with an average of 6.0 ng/m^3^, during the source control period and from 3.1 to 24.5 ng/m^3^, with an average of 11.1 ng/m^3^, during the nonsource control period. We estimated significant BaP_eq_ concentration reductions for all individual PAHs during the source control period relative to the nonsource control period (22–78%) and during the Olympic period relative to the non-Olympic period (32–72%). In particular, estimated ∑PPAH_12-BaPeq_ concentrations were reduced to a greater degree (48% reduction in concentration) than were ∑302PAH_5-BaPeq_ concentrations (38% reduction in concentration). We measured lower ∑PPAH_12-BaPeq_-to-∑302PAH_5-BaPeq_ ratios during the Olympic period than during the non-Olympic period; these may have resulted from more stringent control of coal combustion emissions during the Olympic Games. The BaP_eq_ concentration profile of the individual PAHs was similar among the different periods. We identified BbF, DahA, BaP, and DBalP as the most important carcinogenic PAHs in the Beijing air, which combined contributed to 91–93% of the estimated PAH carcinogenicity. Using a point-estimate approach, we estimated the number of lifetime excess inhalation cancer cases due to exposure to the 17 carcinogenic PAHs to range from 6.5 to 518 per million people for the source control period concentrations and from 12.2 to 964 per million people for the nonsource control period concentrations. This corresponded to a 46% reduction in the estimated inhalation cancer risk and suggests that the cancer risk posed by PAH exposure in Beijing air, as well as air in other Chinese megacities similar to Beijing, can be greatly reduced by effective source control strategies. In addition, the total excess inhalation cancer risk would have been underestimated by 23% if we had not included the five MW 302 PAHs in the estimate. This highlights the importance of including these high-molecular-weight PAHs in future assessments.

## Figures and Tables

**Table 1:** Abbreviations and RPF values of individual PAHs included in the inhalation cancer risk assessment.

PAH	Abbreviation	RPF^a^
Twelve priority pollutant PAHs		
Anthracene	ANT	0
Benz[*a*]anthracene	BaA	0.2
Benzo[*b*]fluoranthene	BbF	0.8
Benzo[*g,h,i*]perylene	BghiP	0.009
Benzo[*k*]fluoranthene	BkF	0.03
Chrysene	CHR	0.1
Dibenz[*a*,*h*]anthracene	DahA	10
Fluoranthene	FLA	0.08
Indeno[1,2,3-*cd*]pyrene	IcdP	0.07
Phenanthrene	PHE	0
Pyrene	PYR	0
Benzo[*a*]pyrene	BaP	1
Five MW 302 PAHs		
Dibenzo[*a*,*l*]pyrene	DBalP	30
Naphtho[2,3-*e*]pyrene	N23eP	0.3
Dibenzo[*a*,*e*]pyrene	DBaeP	0.4
Dibenzo[*a*,*i*]pyrene	DBaiP	0.6
Dibenzo[*a*,*h*]pyrene	DBahP	0.9

a Values taken from U.S. EPA (2010).
